# Anti‐ADAMTS13 Antibodies Trajectory is Associated With ADAMTS13 Recovery in Immune‐Mediated TTP

**DOI:** 10.1002/ajh.70005

**Published:** 2025-07-15

**Authors:** Marie Robert, Arthur Mageau, Ygal Benhamou, François Provôt, Jehane Fadlallah, Lionel Galicier, Elie Azoulay, Hafid Ait‐Oufella, Tomas Urbina, Pascale Poullin, Alain Wynckel, Coralie Poulain, Nihal Martis, Pierre Perez, Virginie Rieu, Yahsou Delmas, Jean‐Michel Halimi, Christelle Barbet, Amélie Seguin, Valérie Chatelet, Jean‐François Augusto, Mathieu Legendre, Olivier Moranne, Carole Philipponnet, Bérengère Cador, Raïda Bouzid, Bérangère S. Joly, Agnès Veyradier, Paul Coppo, the French Reference Center for Thrombotic Microangiopathies, Augusto Jean‐François, Azoulay Elie, Barbay Virginie, Benhamou Ygal, Charasse Christophe, Charvet‐Rumpler Anne, Chauveau Dominique, Ribes Davis, Choukroun Gabriel, Coindre Jean‐Philippe, Coppo Paul, Delmas Yahsou, Kwon Theresa, Salanoubat Célia, Dossier Antoine, Fain Olivier, Ville Simon, Frémeaux‐Bacchi Véronique, Galicier Lionel, Grangé Steven, Guidet Bertrand, Halimi Jean‐Michel, Hamidou Mohamed, Neel Antoine, Fornecker Luc‐Matthieu, Hié Miguel, Jacobs Frédéric, Joly Bérangère, Kanouni Tarik, Kaplanski Gilles, Rieu Claire, Le Guern Véronique, Moulin Bruno, Rebibou Jean‐Michel, Ojeda Uribe Mario, Parquet Nathalie, Pène Frédéric, Perez Pierre, Poullin Pascale, Marie Manon, Presne Claire, Provôt François, Mesnard Laurent, Saheb Samir, Seguin Amélie, Servais Aude, Stépanian Alain, Veyradier Agnès, Vigneau Cécile, Wynckel Alain, Zunic Patricia

**Affiliations:** ^1^ Centre de Référence des Microangiopathies Thrombotiques, Assistance Publique‐Hôpitaux de Paris (AP‐HP) Paris France; ^2^ Département de Médecine Interne Hôpital Bichat‐Claude Bernard, HUPNVS, AP‐HP. Université Paris Cité. Inserm UMR 1137 IAME, Team DESCID Paris France; ^3^ Département de Médecine Interne CHU Charles Nicolle Rouen France; ^4^ Université de Normandie, UNIROUEN, INSERM U1096 EnVI Rouen France; ^5^ Service de Néphrologie Hôpital Albert Calmette Lille France; ^6^ Service d’Immunologie Clinique Hôpital Saint‐Louis, Assistance Publique‐Hôpitaux de Paris (AP‐HP) Paris France; ^7^ Service de Médecine Interne Hôpital La Timone, AP‐HM Marseille France; ^8^ Médecine Intensive Réanimation, Hôpital Saint‐Louis, Assistance Publique‐Hôpitaux de Paris (AP‐HP) Paris France; ^9^ Université Paris‐Cité Paris France; ^10^ Université Paris‐Cité, PARCC, INSERM Paris France; ^11^ Sorbonne Université Service de médecine Intensive‐Réanimation, Assistance Publique, Hôpitaux de Paris (AP‐HP) Paris France; ^12^ Service d’Hémaphérèse CHU Conception Marseille France; ^13^ Service de Néphrologie Hôpital Maison Blanche Reims France; ^14^ Service de Néphrologie, Dialyse et Transplantation Hôpital Universitaire Amiens France; ^15^ Service de Médecine Interne CHU de Nice, Nice, Université Côte d’Azur; Mediterranean Centre for Molecular Medicine, Control of Gene Expression (COdEX), INSERM U1065 Nice France; ^16^ Service de Médecine Intensive Réanimation Hôpital Brabois Nancy France; ^17^ Service de Médecine Interne CHU Estaing Clermont‐Ferrand France; ^18^ Service de Néphrologie CHU Bordeaux Bordeaux France; ^19^ Service de Néphrologie‐Hypertension Artérielle, Dialyses, Transplantation Rénale CHRU Tours Tours France; ^20^ Unité INSERM 1327 Ischemia Université de Tours Tours France; ^21^ Service de Néphrologie‐Immunologie Clinique CHRU de Tours Tours France; ^22^ Service de Réanimation Médicale CHU de Nantes Nantes France; ^23^ Centre Universitaire des Maladies Rénales, CHU de Caen Normandie Caen France; ^24^ Service de Néphrologie‐Dialyse‐Transplantation CHU d’Angers Angers France; ^25^ Service de Néphrologie CHU Dijon Dijon France; ^26^ Service Néphrologie Dialyse Aphérèse Hôpital Universitaire de Nîmes, IDESP Université de Montpellier Montpellier France; ^27^ Service de Néphrologie CHU de Clermont‐Ferrand Clermont‐Ferrand France; ^28^ Service de médecine Interne et Immunologie Clinique CHU de Rennes Rennes France; ^29^ INSERM Unité Mixte de Recherche S (UMRS) 1138, Centre de Recherche des Cordeliers Paris France; ^30^ Service d’Hématologie Biologique Hôpital Lariboisière, AP‐HP Paris France; ^31^ Service d’Hématologie AP‐HP, Sorbonne Université Paris France

**Keywords:** ADAMTS13, caplacizumab, immune‐mediated thrombotic thrombocytopenic purpura, immunosuppression, prognosis, rituximab

## Abstract

Current triplet regimens associating therapeutic plasma exchange (TPE), immunosuppression with corticosteroids and rituximab, and caplacizumab have dramatically improved the outcome of immune‐mediated thrombotic thrombocytopenic purpura (iTTP). However, nearly half of the patients require extended caplacizumab treatment (i.e., > 30 days) due to persistent ADAMTS13 deficiency, raising cost and tolerance concerns. Therefore, we investigated whether anti‐ADAMTS13 antibodies titer and their trajectory during the acute phase of the disease could predict ADAMTS13 improvement (i.e., activity ≥ 20% before day‐30 post‐TPE). From a cohort of 286 patients receiving the triplet regimen, we identified on diagnosis a cut‐off value for anti‐ADAMTS13 IgG antibodies of 90.5 U/mL, with a modest discriminating ability (AUC: 0.57) for predicting long‐term response, precluding its use to guide therapeutic strategies. Nonetheless, the analysis of anti‐ADAMTS13 IgG antibodies titer trajectory from diagnosis revealed that the proportion of iTTP patients with ADAMTS13 activity improvement was higher in patients who decreased (Dec+) their antibodies titer within the 7–14 days interval post‐TPE compared to those without decrease (Dec−) (65% vs. 25% of cases, respectively, *p* < 0.001), a finding confirmed in a validation cohort (*N* = 51). These results highlight the possibility of intensifying immunosuppression in an early period post‐TPE to shorten time to ADAMTS13 activity recovery. Close monitoring of anti‐ADAMTS13 antibodies titer may guide immunomodulation strategies, including additional courses of B‐cell depleting agents when appropriate.

## Graphical abstract

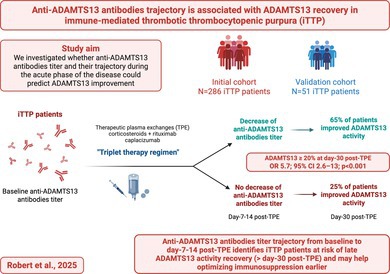

## 1 Introduction

Immune‐mediated thrombotic thrombocytopenic purpura (iTTP) is a severe disorder caused by a profound, IgG antibody‐mediated, deficiency in the Von Willebrand factor (VWF)‐cleaving protease ADAMTS13 (*A disintegrin and metalloprotease with thrombospondin type 1 repeats, member 13*). As a result, uncleaved ultra‐large multimers of VWF accumulate, causing excessive platelet clumping, generalized microvascular occlusion, along with microangiopathic hemolytic anemia and severe platelet consumption [1, 2]. Although previously associated with a high mortality rate [3], the prognosis of iTTP has significantly improved through the systematic use of therapeutic plasma exchange (TPE) and corticosteroids. Over the last few years, rituximab, a B‐cell depleting agent, and more recently caplacizumab, a first‐in‐class anti‐adhesive nanobody directed against the A1 domain of VWF, have been successfully associated with TPE [4, 5, 6], allowing recovery rates exceeding 95% [1, 7]. As the efficacy of rituximab is typically observed at least 2 weeks following the first administration [8, 9, 10], caplacizumab is continued for 30 days after clinical response (i.e., after the last TPE) to provide a bridge to antibody depletion and ADAMTS13 recovery [4, 11]. However, nearly half of the patients require caplacizumab beyond this period when ADAMTS13 levels remain persistently undetectable [4, 5], thus raising concerns for cost‐effectiveness and tolerance [12, 13, 14]. Such situations may require intensified immunosuppression to meet long‐term remission. Identifying predictors of ADAMTS13 recovery could therefore help shorten the time to ADAMTS13 improvement in iTTP patients and reduce prolonged caplacizumab use.

In this study, we analyzed iTTP patients systematically treated with a “triplet therapy” regimen associating TPE, immunosuppression (i.e., corticosteroids and rituximab) and caplacizumab [5]. Our objective was to determine whether anti‐ADAMTS13 IgG antibodies titer and/or their trajectory during the acute phase of the disease could help identify early long‐term responders and guide targeted immunosuppression strategies.

## 2 Methods

### 2.1 Study Design

To identify predictive features associated with delayed ADAMTS13 activity improvement in iTTP patients, we first compared at baseline patients who improved ADAMTS13 activity ≥ 20% (considered as a protective activity for iTTP) [15, 16] between 7 and 30 days post‐TPE (termed hereafter rapid responders) to those who improved ADAMTS13 activity ≥ 20% after day 30 post‐TPE, and referred hereafter late responders. Secondly, we assessed whether the early trajectory of anti‐ADAMTS13 IgG antibodies titer from baseline to day 7 or day 14 post‐TPE, considered as a possible time point to intensify immunosuppressive treatment, could predict time to ADAMTS13 activity improvement at day 30 post‐TPE. To this aim, we compared patients who experienced a decrease (Dec+ group) in the anti‐ADAMTS13 IgG antibodies titer between baseline and day 7 or day 14 post‐TPE interruption, to those with a stable or an increase (Dec− group) in anti‐ADAMTS13 IgG antibodies titer during the same frame time. Anti‐ADAMTS13 IgG antibodies were not further assessed when ADAMTS13 activity reached ≥ 20%. Patients without a quantitative anti‐ADAMTS13 IgG antibodies titer were not included in this analysis.

### 2.2 Patients

Adult patients with a clinical diagnosis of iTTP were prospectively enrolled in the registry of the French Reference Center for Thrombotic Microangiopathy (CNR‐MAT) network from September 2018 (date of caplacizumab availability for iTTP in France) to July 2023. To validate our results on a prospective series of patients, a second group of patients (validation group) managed with the same conditions was set up from the same centers during the analysis period of the study group (i.e., from August 2023 to August 2024). Diagnosis of iTTP required findings of thrombotic microangiopathy with ADAMTS13 activity < 10% and anti‐ADAMT13 IgG titers ≥ 15 U/mL [17]. Patients with associated conditions such as pregnancy, sepsis, flare‐ups of systemic autoimmune disease, organ transplantation, and malignancy were excluded. To work on a homogeneous group of patients, only patients who had received a standardized “triplet therapy” regimen defined by daily TPE, immunosuppression (i.e., frontline rituximab and corticosteroids) and caplacizumab were included in this study [5]. Clinical data were collected through a standardized form at baseline and during follow‐up.

### 2.3 Treatment and Outcome

Daily TPE were performed until clinical response was reached. Corticosteroids were usually started at a prednisone‐equivalent dose of 1 mg/kg/day [maximal dose, 100 mg/day] and tapered for a total of 3 weeks. Rituximab was administered intravenously on day 1 of TPE, and then days 4, 8, and 15 at a dose of 375 mg/m^2^, as validated [9, 10, 18]. Caplacizumab was initiated with an intravenous loading dose of 10 mg prior to the first TPE, and subsequently administered as a 10 mg subcutaneous dose after each TPE, usually for a 30‐day period after TPE interruption [18]. In some patients, caplacizumab was stopped before day 30 post‐TPE if ADAMTS13 activity assessed weekly post‐TPE reached ≥ 20%, as per practitioner's choice. Following the 30‐day period post‐TPE, caplacizumab could be extended for up to four additional weeks if ADAMTS13 activity remained depressed, or until partial ADAMTS13 recovery (defined by the achievement of an activity ≥ 20%–< 50%) [15] regardless of the time required, as per practitioner's choice.

Responses to treatment were assessed according to previously described criteria [15]. Clinical response was defined as a sustained platelet count recovery (≥ 150 × 10^3^/μL). Following clinical response and TPE interruption, ADAMTS13 activity was assessed weekly until durable complete recovery (activity ≥ 50%) [15] as per national recommendation (https://www.has‐sante.fr/upload/docs/application/pdf/2022‐10/pnds_ptt_26092022_final.pdf; page 61, in French). iTTP severity was assessed using a severity score, the French Severity Score, based on cerebral involvement (including confusion, stupor, coma or focal deficiency), age, and lactate dehydrogenase (LDH) > 10 times the upper normal value level that reflects primarily end organ injury. Patients were then classified into three groups based on their risk of early death [3].

### 2.4 ADAMTS13 Activity and Anti‐ADAMTS13 IgG Titer

In most cases, ADAMTS13 activity was assessed using the reference method (FRETS‐VWF73) as previously described [5, 19]. In all centers, anti‐ADAMTS13 IgG antibodies were quantified with enzyme‐linked immunosorbent assay (ELISA) using the TECHNOZYM ADAMTS13 INH ELISA assay (Technoclone, Vienna, Austria), following the manufacturer's guidelines [5]. The threshold for positivity for anti‐ADAMTS13 IgG was set at 15 U/mL. Patients with other methods used to detect anti‐ADAMTS13 antibodies were excluded. The turnaround time for obtaining anti‐ADAMTS13 IgG antibodies titer is usually 7 days, although for emergencies (e.g., iTTP patients with pregnancy), it can be expedited to less than 3 days [20, 21]. The search for a plasma inhibitor of ADAMTS13 was not included in the analysis as it was shown that the main pathogenic mechanism in iTTP relies on the autoantibody‐mediated ADAMTS13 clearance instead of ADAMTS13 inhibition [22].

### 2.5 Statistics

Quantitative data are represented as median (Interquartile range, IQR) and categorical data as number (percentages). Data were compared between iTTP patients according to the completion of the main outcome using Fisher's exact tests, Pearson's Chi‐squared tests, and Wilcoxon rank sum tests, when appropriate. Receiver operating characteristic (ROC) curves were plotted using the absence of ADAMTS13 activity recovery as the outcome and an elevated titer of anti‐ADAMTS13 antibodies as the exposure. Confidence intervals (CI) of the area under the ROC curve and of the diagnostic performances of the different thresholds were determined by bootstrapping (1000 iterations). The cumulative incidence function was used to plot the cumulative proportion of patients achieving ADAMTS13 activity recovery over time. Univariable and multivariable logistic regression models were used to calculate the odds ratio (OR) of parameters of interest associated with ADAMTS13 recovery. All tests were two‐sided; *p* < 0.05 was considered statistically significant. Analyses were carried out using R software (version 4.3.1).

## 3 Results

### 3.1 Population Characteristics and iTTP Presentation

Of the 395 patients with iTTP identified over the 58‐month period, 286 patients were ultimately included in the analyses, whereas 109 others were excluded due to the use of an alternative anti‐ADAMTS13 antibody assay (*n* = 50); incomplete inclusion criteria (*n* = 53), and absence of ADAMTS13 activity assessment within 30 days post‐TPE (*n* = 6) (flow‐chart detailed in Figure S1).

### 3.2 Clinical Features According to Kinetics of ADAMTS13 Recovery

ADAMTS13 activity ≥ 20% was achieved after a median time of 25 days (IQR, 15–47) in the whole cohort. One hundred and sixty‐one patients (56.3%) achieved ADAMTS13 activity ≥ 20% between day 7 and day 30 post‐TPE, within a median of 17 days post‐TPE (IQR, 11–23), and were considered as rapid responders. Conversely, 125 patients (43.7%) achieved an ADAMTS13 activity ≥ 20% after day 30 post‐TPE, within a median time of 54 days (IQR, 39–84.5) post‐TPE (i.e., late responders), with 57 patients (20% among the whole cohort) requiring more than 58 days to achieve an ADAMTS13 activity ≥ 20%. Of note, a patient who never achieved ADAMTS13 recovery during the study period was included in the late responder group.

Patients' characteristics from both groups were similar at baseline, but late responders had a higher median anti‐ADAMTS13 IgG antibody titer than rapid responders (*p* = 0.028) (Table 1). The median number of TPE sessions and the use of corticosteroids were similar between groups; expectedly, late responders received more rituximab infusions than rapid responders (*p* = 0.009) and required longer periods of caplacizumab post‐TPE (*p* < 0.001) (Table 1). Three patients from the late responder group received salvage therapy (twice‐daily TPE, *n* = 2; vincristine, *n* = 1). No additional immunosuppressive agents than corticosteroids and rituximab were used in the acute phase of the disease. Five patients (1.7%), all from the late responder group, died within the first 3 months subsequent to iTTP diagnosis (Table 1). Two deaths were directly related to iTTP (a severe bilateral pulmonary embolism leading to cardiac arrest in one case, and neurologic complications of iTTP in the other), whereas in the three others, death was related to COVID‐19 (*n* = 2) and a massive hemorrhage following bone marrow aspirate. One patient from each group presented with refractory disease; nine patients (7.2%) from the late responder group, and six patients (3.7%) from the rapid responder group presented with an exacerbation under caplacizumab (*p* = 0.2) (Table 1). Exacerbations occurred in the context of infection (*n* = 6, COVID‐19, flu, viral infection, pyelonephritis, septic shock) or because treatment was alleviated too early (*n* = 5, mostly in context of associated thrombosis requiring anticoagulation).

**TABLE 1:** Clinical features at baseline and outcome according to the primary outcome.

	Day‐30 ADAMTS13 < 20% post‐TPE (*N* = 125)	Day‐30 ADAMTS13 ≥ 20% post‐TPE (*N* = 161)	*p*
Characteristics at baseline			
Age (y)	46 [34–57]	41 [32–53]	0.10
Female gender	86 (69)	107 (66)	0.7
Body mass index (kg/m^2^)	26.3 [23.5–30.3]	26.9 [22.3–31.9]	> 0.9
Ethnicity			
White	86 (70)	127 (79)	0.2
African‐West Indies	30 (24)	28 (18)	
Asian	7 (5.7)	5 (3.1)	
History of iTTP	10 (8.1)	21 (13)	0.2
Cardiac involvement	49 (39)	53 (33)	0.3
Neurologic involvement	82 (66)	107 (67)	0.8
Platelet count (× 10^3^/μL)	12 [8–20]	12 [8–18]	0.5
Hemoglobin (g/dL)	8.3 [7–9.7]	8.4 [6.6–9.6]	0.4
Serum creatinine (μmol/L)	93 [68–120]	96 [74–126]	0.3
LDH level xN	3.9 [2.2–5.6]	4 [2.7–6.2]	0.4
ADAMTS13 activity (%)	< 10	< 10	—
Anti‐ADAMTS13 IgG Abs titer (U/mL)	79 [46–112]	66 [40–93]	0.028
Anti‐ADAMTS13 IgG Abs titer categories (U/mL)			
15–90.5	71 (57)	118 (73)	0.003
> 90.5	54 (43)	43 (27)	
French severity score			
Low	72 (58)	105 (65)	0.3
Intermediate	34 (27)	39 (24)	
High	19 (15)	17 (11)	
Treatment			
TPE sessions to clinical response	4 [4–6]	4.5 [4–6]	0.7
Corticosteroids	125 (100)	161 (100)	0.6
Rituximab	125 (100)	161 (100)	0.3
< 4 infusions	19 (15)	39 (24)	0.009
4 infusions	91 (73)	116 (72)	
> 4 infusions	15 (12)	6 (3.8)	
Caplacizumab post‐TPE (days)	41.5 [31–69]	26 [20–30]	< 0.001
Outcome			
Clinical response	123 (98)	159 (99)	0.6
Time to clinical response from first TPE (days)	4 [3–5.5]	4 [3–5]	0.7
Time from diagnosis to ADAMTS13 ≥ 20% (days)	60 [42–85.8]	22 [15–28]	< 0.001
Time to ADAMTS13 ≥ 20% post‐TPE (days)	54 [39–84.5]	17 [11–23]	< 0.001
Refractoriness	1 (0.8)	1 (0.6)	> 0.9
Exacerbation during caplacizumab therapy	9 (7.2)	6 (3.7)	0.2
Salvage therapy	3 (2.4)	0	0.082
3‐month death rate	5 (4)	0	0.01

*Note*: Data are given as median (25th–75th percentile) for quantitative variables and as *n* (%) for qualitative variables. Severe ADAMTS13 deficiency was defined as an activity < 10% (normal range for ADAMTS13 activity 50%–100%). The positivity threshold of anti‐ADAMTS13 IgG antibodies (Abs) was 15 U/mL, according to the manufacturer's instructions (Technoclone). Cardiac involvement was defined as the presence of clinical manifestations (e.g., chest pain) and/or electrocardiographic abnormalities. Neurologic involvement included the occurrence of confusion and/or stupor and/or seizures and/or coma and/or focal deficiency. Patients at high risk of early death of iTTP were defined by a French severity score ≥ 3 (cerebral involvement: yes = 1/no = 0, LDH: > 10xULN = 1/≤ 10xULN = 0, age: > 60 y = 2/< 40 and ≤ 60 y = 1/≤ 40 y = 0). Cerebral involvement included confusion, stupor, coma or focal deficiency [3].

Abbreviations: Abs, antibodies; ADAMTS13, A Desintegrin And Metalloproteinase with ThromboSpondin‐1 motifs, 13rd member; iTTP, immune‐mediated thrombotic thrombocytopenic purpura; LDH, lactate dehydrogenase; TPE, therapeutic plasma exchange.

### 3.3 Clinical Presentation According to Baseline Anti‐ADAMTS13 IgG Titers

As anti‐ADAMTS13 IgG antibody titers differed between rapid and late responder groups, we first aimed to determine whether a threshold of anti‐ADAMTS13 IgG antibody titer at iTTP diagnosis would be predictive of ADAMTS13 activity recovery at 30 days post TPE. A baseline anti‐ADAMTS13 IgG antibody titer of 90.5 U/mL was identified as a threshold value predictive of ADAMTS13 activity recovery at day 30 post‐TPE, using ROC curve analysis (Figure S2). The sensitivity of this threshold was 0.43 (95% CI, 0.34–0.52) and its specificity 0.73 (95% CI, 0.66–0.80). The positive predictive value (i.e., the proportion of patients with anti‐ADAMTS13 IgG antibodies titer > 90.5 U/mL at diagnosis and without ADAMTS13 ≥ 20% at day‐30 post‐TPE) was 0.56 (95% CI, 0.46–0.65) and the negative predictive value (i.e., the proportion of patients with anti‐ADAMTS13 IgG antibodies titer ≤ 90.5 U/mL at diagnosis and with ADAMTS13 ≥ 20% at day‐30 post‐TPE) was 0.62 (95% CI, 0.55–0.69). The area under the curve (AUC) was 0.57 (95% CI, 0.51–0.64) (Figure S2).

**FIGURE 1:**
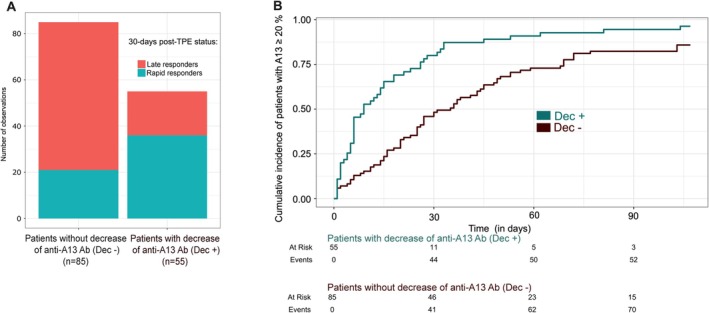
ADAMTS13 recovery at day 30 post‐TPE cessation according to the trajectory of anti‐ADAMTS13 IgG antibodies titer between diagnosis and day‐7 to day‐14 post‐TPE. Proportions of iTTP patients with ADAMTS13 recovery at day 30 post‐TPE cessation according to the evolution of anti‐ADAMTS13 IgG antibodies titer (A). Cumulative incidence curve of patients with ADAMTS13 recovery according to the evolution of anti‐ADAMTS13 IgG antibodies titer (B). T_0_ corresponds to day 14 post‐TPE cessation. Abbreviations: ADAMTS13, A Disintegrin And Metalloproteinase with ThromboSpondin‐1 motifs, 13rd member; Dec + and Dec‐ denotes patients who decreased (+) or not (−) their anti‐ADAMTS13 IgG antibodies titer between diagnosis and day 7 to day 14 post‐TPE; TPE: Therapeutic plasma exchange. [Color figure can be viewed at wileyonlinelibrary.com]

Baseline presentation was comparable between iTTP patients regardless of their anti‐ADAMTS13 IgG titers, except for creatinine and LDH levels, which were higher in patients with anti‐ADAMTS13 IgG antibody titers > 90.5 U/mL. Patients with baseline anti‐ADAMTS13 IgG antibodies > 90.5 U/mL required a greater number of TPE sessions to achieve clinical response (*p* = 0.03), a longer time to achieve an ADAMTS13 activity ≥ 20% from baseline (*p* = 0.013), and accordingly, more rituximab infusions (Table S1 and Figure 1A). Similarly, time to ADAMTS13 activity ≥ 20% post‐TPE was longer in patients with baseline anti‐ADAMTS13 IgG antibodies > 90.5 U/mL (*p* = 0.004) (Table S1, Figure S3).

### 3.4 Trajectories of Anti‐ADAMTS13 IgG Titers and ADAMTS13 Recovery

As the identification of a threshold of anti‐ADAMTS13 IgG antibodies titer at iTTP diagnosis poorly predicted ADAMTS13 activity improvement at day 30 post‐TPE, we next sought whether the early trajectory of anti‐ADAMTS13 IgG antibodies titer between baseline and day 7 or day‐14 days post‐TPE had a better predictive value of ADAMTS13 activity ≥ 20% at 30‐day post‐TPE. To this end, we compared patients with a decrease (Dec+) to those with an absence of decrease (i.e., stable or increase) (Dec−) in anti‐ADAMTS13 IgG antibodies titer during this period of time and independently from the cut‐off (i.e., 90 U/mL) identified previously. Among the whole population of 286 patients, 140 patients with available data on anti‐ADAMTS13 IgG antibodies still had a severe ADAMTS13 deficiency (activity < 20%) at day 7 or day 14 post‐TPE, while the remaining patients either had already reached ADAMTS13 recovery (*n* = 68) and/or did not have available data regarding anti‐ADAMTS13 titers during follow‐up (*n* = 78).

The Dec+ group included 55/140 patients (39%), whereas the remaining 85 patients (61%) made up the Dec− group (Table 2). At baseline, clinical features were not significantly different between Dec+ and Dec− groups, even in regard to anti‐ADAMTS13 IgG antibodies titers. Patients of both groups were equally treated (i.e., rituximab use, corticosteroids, delay in initiating caplacizumab). Clinical response was achieved within 4 days in both groups. We observed a greater proportion of rapid responders in the Dec+ group (65%) than in the Dec− group (25%) (OR = 5.7 [95% CI, 2.6–13], *p* < 0.001) (Figure 1A). The median duration of caplacizumab treatment post‐TPE was longer for Dec− patients than for Dec+ patients (41 days [IQR, 30–68.5] vs. 28 days [IQR, 22.5–31], *p* < 0.001). Moreover, the cumulative incidence of ADAMTS13 activity ≥ 20% at day‐30 post‐TPE was higher in the Dec+ group than in Dec− patients (Log‐rank test, *p* < 0.001; Figure 1B and Table 2). Clinical response, refractoriness, 3‐month survival, and exacerbations did not differ between groups. One patient from the Dec− group received salvage therapy (i.e., twice‐daily TPE). The sensitivity of Dec+ status in predicting rapid response was 0.77 (95% CI, 0.67–0.88) and its specificity 0.63 (95% CI, 0.51–0.75). The predictive positive value was 0.75 (95% CI, 0.73–0.86), and the negative predictive value was 0.65 (95% CI, 0.60–0.70). By multivariate analysis, we could confirm that anti‐ADAMTS13 IgG antibodies trajectory represented an independent predictive value over confounding variables associated with a rapid ADAMTS13 response (Table S2).

**TABLE 2:** Clinical features at baseline and outcomes according to anti‐ADAMTS13 antibodies titer trajectory.

	Dec− group (*N* = 85)	Dec+ group (*N* = 55)	*p*
Characteristics at baseline			
Age (y)	47 [37–54]	44 [31–58.5]	0.6
Female Sex	61 (72)	36 (65)	0.4
Body mass index	26.3 [23.5–30.3]	27.2 [22.6–33.1]	0.8
Ethnicity			
White	66 (78)	36 (68)	0.12
African‐West Indies	18 (21)	13 (25)	
Asian	1 (1.2)	4 (7.5)	
History of iTTP	10 (12)	4 (7.3)	0.4
Cardiac involvement	31 (36)	27 (49)	0.14
Neurological involvement	61 (72)	37 (67)	0.6
Platelet count (× 10^3^/μL)	11 [8–18]	12 [8–18]	0.6
Hemoglobin (g/dL)	8.4 [7–9.9]	8.5 [6.9–9.5]	> 0.9
Serum creatinine level (μmol/L)	85 [65.9–115]	99.5 [74–135.5]	0.09
LDH level xN (U/L)	4.4 [3–6]	4.1 [3–6.3]	0.8
ADAMTS13 activity (%)	< 10	< 10	—
Anti‐ADAMTS13 IgG Abs titer (U/mL)	71 [41–105]	74 [55–95]	0.4
Anti‐ADAMTS13 IgG Abs titer categories (U/mL)			
15–90.5	53 (62)	34 (62)	> 0.9
> 90.5	32 (38)	21 (38)	
French severity score			
Low	47 (55)	32 (58)	> 0.9
Intermediate	26 (31)	16 (29)	
High	12 (14)	7 (13)	
Treatment			
TPE sessions to clinical response	4 [3–5]	5 [4–6]	0.2
Corticosteroids	85 (100)	55 (100)	> 0.9
Rituximab	85 (100)	55 (100)	> 0.9
< 4 infusions	13 (16)	11 (20)	0.8
4 infusions	64 (75)	40 (73)	
> 4 infusions	8 (9.6)	4 (7.3)	
Caplacizumab post‐TPE (days)	41 [30–68.5]	28 [22.5–31]	< 0.001
Outcome			
Clinical response	85 (100)	55 (100)	—
Time to clinical response from first TPE (days)	4 [3–6]	4 [3–5]	> 0.9
Refractoriness	0	0	—
Exacerbation during caplacizumab therapy	6 (7.1)	2 (3.6)	0.5
Salvage therapy	1 (1.2)	0	> 0.9
Time from diagnosis to ADAMTS13 activity ≥ 20% (days)	51 [37–72.3]	28 [21–39]	< 0.001
ADAMTS13 activity ≥ 20% before 30‐day post‐TPE	21 (25)	36 (65)	< 0.001
Time to ADAMTS13 ≥ 20% post‐TPE (days)	49 [30–83]	23 [18.5–40]	< 0.001
3‐month death rate	1 (1.2)	0	> 0.9

*Note*: Dec+ and Dec− denote patients who decreased (+) or did not (−) their anti‐ADAMTS13 IgG antibodies titer between diagnosis and day‐7 to day‐14 post‐TPE. Data are given as median (25th–75th percentile) for quantitative variables and as *n* (%) for qualitative variables. Severe ADAMTS13 deficiency was defined as an activity < 10% (normal range for ADAMTS13 activity 50%–100%). The positivity threshold of anti‐ADAMTS13 IgG antibodies (Abs) was 15 U/mL, according to the manufacturer's instructions (Technoclone). Cardiac involvement was defined as the presence of clinical manifestations (e.g., chest pain) and/or electrocardiographic abnormalities. Neurologic involvement included the occurrence of confusion and/or stupor and/or seizures and/or coma and/or focal deficiency. Patients at high risk of early death from iTTP were defined by a French severity score ≥ 3 (cerebral involvement: yes = 1/no = 0, LDH: > 10xULN = 1/≤ 10xULN = 0, age: > 60 years = 2/< 40 and ≤ 60 years = 1/≤ 40 years = 0). Cerebral involvement included confusion, stupor, coma, or focal deficiency [3]. Clinical response corresponds to platelet count recovery (≥ 150 × 10^3^/μL).

Abbreviations: ADAMTS13, A Disintegrin And Metalloproteinase with ThromboSpondin‐1 motifs, 13rd member; iTTP, immune‐mediated thrombotic thrombocytopenic purpura; LDH, lactate dehydrogenase; TPE, therapeutic plasma exchange.

### 3.5 Accuracy and Validation of Early Anti‐ADAMTS13 Antibodies Trajectory as a Predictor of ADAMTS13 Recovery

To confirm our results on the association between the trajectory of anti‐ADAMTS13 IgG antibodies titers and ADAMTS13 recovery, we set up a validation cohort of 51 iTTP patients. The Dec+ group included 22/51 patients (43%), whereas the remaining 29 patients (57%) made up the Dec− group (detailed in Table S3). Baseline anti‐ADAMTS13 IgG antibodies titers were not significantly different between Dec+ and Dec− groups. We first confirmed that rapid responders were more prevalent in the Dec+ group (59%) than in the Dec‐ group (0%) (*p* < 0.001) (Figure S4A). As expected, the median duration of caplacizumab treatment post‐TPE was longer for Dec− patients than for Dec+ patients (58 days [IQR, 42.8–77.3] vs. 36 days [IQR, 27.3–53.3], *p* = 0.035) (Table S3). We confirmed that the cumulative incidence of ADAMTS13 activity ≥ 20% at day‐30 post‐TPE was higher in the Dec+ group than in Dec− patients (Log‐rank test, *p* < 0.001; Figure S4B and Table 3). Clinical response, refractoriness, 3‐month survival, and exacerbations were similar between groups (Table 3).

**TABLE 3:** Outcomes according to anti‐ADAMTS13 antibodies titer trajectory in the validation cohort.

	Dec− group (*N* = 29)	Dec+ group (*N* = 22)	*p*
Clinical response	29 (100)	21 (95)	0.4
Time to clinical response from first TPE (days)	4 [3–4]	3 [2–5]	0.3
Refractoriness	0	1 (4.5)	0.4
Exacerbation during caplacizumab therapy	2 (6.9)	1 (4.5)	> 0.9
Time from diagnosis to ADAMTS13 activity ≥ 20% (days)	76 [56–112]	36 [28–45]	< 0.001
ADAMTS13 activity ≥ 20% before 30‐day post‐TPE	0	13 (59)	< 0.001
Time to ADAMTS13 ≥ 20% post‐TPE (days)	74 [53–103]	27 [21.3–38.5]	< 0.001
3‐month death rate	0	0	—

*Note*: Dec+ and Dec− denotes patients who decreased (+) or not (−) their anti‐ADAMTS13 IgG antibodies (Abs) titer between diagnosis and day 7 to day 14 post‐TPE. Data are given as median (25th–75th percentile) for quantitative variables and as *n* (%) for qualitative variables. Clinical response corresponds to platelet count recovery (≥ 150 × 10^3^/μL).

Abbreviations: ADAMTS13, A Disintegrin And Metalloproteinase with ThromboSpondin‐1 motifs, 13rd member; TPE, therapeutic plasma exchange.

## 4 Discussion

We report here the analysis of anti‐ADAMTS13 IgG antibodies titer trajectory from baseline to day 7–14 post‐TPE as a reliable approach to identify iTTP patients at risk of late response to the triplet therapy regimen. Late response, as defined here by an improvement of ADAMTS13 activity ≥ 20% beyond day‐30 post‐TPE, was a common condition affecting 44% of patients, consistent with our preliminary results [5]. In the more severe cases, ADAMTS13 activity improvement was delayed beyond day‐58 post‐TPE, exposing patients to a prolonged treatment with caplacizumab and raising concerns about tolerance but also cost‐effectiveness [14]. So far, attempts to alleviate the burden of caplacizumab treatment post‐TPE only consisted in the prolongation of the treatment to every other day during its extension period [23]. The prompt identification of these patients through our strategy could result in an original approach consisting in the adjustment of the immunosuppressive regimen with additional courses of rituximab or other B‐cell depleting strategies [24, 25, 26, 27] shortly after TPE termination, typically by day‐7 or day‐14 post‐TPE once IgG antibody titers are available, to improve ADAMTS13 activity within the 2 weeks period (i.e., by day‐30 post‐TPE) corresponding to the time of efficacy of anti‐B‐cell agents [8, 10]. Conversely, the use of a baseline anti‐ADAMTS13 titer threshold of 90.5 U/mL had a modest ability to discriminate rapid from late responders, precluding its use in early decision‐making. Our results support preliminary works carried out, where longitudinal assessment of anti‐ADAMTS13 IgG antibodies titers within 3 to 7 days after TPE initiation was suggestive of iTTP exacerbation or recurrence [28, 29].

Previous work has examined anti‐ADAMTS13 IgG antibodies as prognostic markers to intensify treatment and hasten remission. Higher anti‐ADAMTS13 IgG antibodies titers with lower ADAMTS13 antigens levels at baseline were associated with higher mortality, especially when anti‐ADAMTS13 IgG antibodies are in the upper quartile and ADAMTS13 antigens in the lowest quartile [30]. At clinical response or remission, these features also predicted exacerbation or recurrence [28]. In accordance with previous reports, our study found that anti‐ADAMTS13 titers at diagnosis offer only limited utility for predicting long‐term response, precluding their use to guide therapeutic strategies [28, 29]. Nonetheless, we show that the decrease in anti‐ADAMT13 IgG antibody titers could be a reliable predictor to discriminate rapid from late responders with a positive predictive value of 75% and a negative predictive value of 65%. These results were validated in a second cohort, which strengthens our conclusions. Whether other markers [23] could be used in combination with anti‐ADAMTS13 IgG antibody trajectory to better refine the duration of caplacizumab treatment in iTTP needs further investigations. In the present study, we selected two timepoints to measure IgG anti‐ADAMTS13 titers: a point at baseline and a point at day 7–14 post‐TPE. We set a minimum of 7 days after TPE because TPE can significantly alter IgG measurements, lowering serum IgG by about 63% after one session and up to 90% after 3 days [31]. Still, limited data also suggest that extracorporeal IgG removal might trigger extra‐antibody production, resulting in rising and ultimately higher antibody levels post‐TPE [32]. Lastly, results of anti‐ADAMTS13 antibodies can usually be available 5–7 days after sampling [20]. Since the median number of TPE sessions in our cohort was approximately four in our study, the 7–14‐day interval post‐TPE roughly corresponds to day 15 after TTP diagnosis, with results available around day 21. One central aim of our study was to identify patients whose ADAMTS13 activity would take more than 30 days to recover; thus, these two time points provided the most informative assessment for guiding potential therapeutic adjustments (e.g., the use of anti‐B‐cell agents). These considerations justify why no additional time points were investigated.

A delayed recovery of ADAMTS13 activity has recently been documented in patients treated with caplacizumab‐based regimens, with failures to achieve ADAMTS13 activity > 30% within 56 days post‐TPE that were six times more prevalent than in historical cohorts [13]. The reason for a delayed normalization of ADAMTS13 is still unclear, although a lower clearance of anti‐ADAMTS13 antibodies resulting from a reduced number of TPE sessions has been advocated [5]. Although still debated [33], this finding suggests that current caplacizumab‐based regimens [34, 35] could expose patients to a substantial delay in immunological remission. However, the benefit of the triplet regimen has been reinforced in 1015 iTTP patients treated with caplacizumab as compared to 510 iTTP patients treated without caplacizumab [36]. Nonetheless, this finding further supports the need to identify patients at risk of long‐term response early in order to optimize immunosuppression. Based on antibodies titer trajectory, treatment could be intensified with additional cycles of rituximab; alternatively, second generation B‐cell depleting agents could be proposed as encouraging results were observed in patients unresponsive to rituximab [27, 37].

The major limitation of our study lies in the retrospective collection of anti‐ADAMTS13 antibody titers, excluding 13% of patients. However, the systematic monitoring of patients receiving a caplacizumab‐based regimen and the relevant time points for ADAMTS13 activity assessment were pre‐defined consensually by a national committee of experts [5], supporting consistency in the exploration of our patients. Moreover, our work only included patients who had an assessment of anti‐ADAMTS13 IgG antibody titers [38] and we believe that our methodology is robust and our results solid, as validated in a second cohort. Our relatively small cohort size prevented us from conducting deeper correlations between the magnitude of the variations of anti‐ADAMTS13 IgG antibody titers and ADAMTS13 recovery. Nonetheless, for practical clinical purposes, we considered that identifying patients into two groups (Dec+ and Dec−) would be more feasible than assessing patients individually according to anti‐ADAMTS13 IgG levels. In addition, our results cannot be applied to patients with acquired TTP without detectable anti‐ADAMTS13 antibodies at baseline (also termed uTTP) However, these patients may achieve ADAMTS13 activity > 20% faster than those with detectable autoantibodies [20, 21], and therefore may not require prolonged caplacizumab treatment.

We provide here evidence that the analysis of anti‐ADAMTS13 IgG antibodies titer trajectory within an interval of 7–14 days post‐TPE, by identifying late responders to the triplet regimen, should help optimize immunosuppression earlier to allow a faster recovery of ADAMTS13 activity by Day 30 post‐TPE, shorten caplacizumab treatment duration, and alleviate the burden of care in these iTTP patients. Prospective studies are now required to strengthen these results and address the feasibility of this strategy.

## Author Contributions

M.R. collected the data, participated in the design of the study, and wrote the first version of the manuscript. A.M. participated in the design of the study and performed the statistical analysis. Y.B., F.P., J.F., L.G., E.A., H.A.O., T.U., P.Po., A.W., C.P., N.M., P.Pe., V.R., Y.D., J.M.H., C.B., A.S., V.C., J.F.A., J.M.R., O.M., and C.P. enrolled patients and collected clinical and laboratory information. R.B. collected the data and prepared the data file for statistical analyses. B.S.J. and A.V. participated in the design of the study and studied ADAMTS13 activity and anti‐ADAMTS13 IgG antibodies titers. P.C. enrolled patients, participated in the design of the study, edited the manuscript extensively, and conducted the work. N.M. provided critical and language editing. All the authors critically reviewed and substantially improved the manuscript.

## Ethics Statement

This study was part of the Thrombotic Microangiopathy program study approved by our institutional review board (CPP04807) in accordance with the Declaration of Helsinki and the French Data Protection Authority.

## Conflicts of Interest

Paul Coppo is a member of the Clinical Advisory Board for Alexion, Sanofi, and Takeda. Pascale Poullin is a member of an Advisory board for Sanofi. Agnès Veyradier is a member of Advisory boards for Sanofi and Takeda. Bérangère S. Joly is a member of an Advisory board for Takeda. Ygal Benhamou has participated in Advisory boards for Sanofi. The remaining authors declare no competing financial interests.

## Supporting information


**Figure S1. Study flowchart**.
Abbreviations: ADAMTS13: A Desintegrin And Metalloproteinase with ThromboSpondin‐1 motifs, 13rd member; iTTP: immune‐mediated thrombotic thrombocytopenic purpura; TPE: therapeutic plasma exchange. *Three patients were not treated with TPE, 4 patients had an ADAMTS13 activity > 10%, and 46 patients had anti‐ADAMT13 IgG antibodies titer < 15 U/mL.

**Figure S2. ROC (Receiver Operating Characteristic) curve showing diagnosis accuracy of initial titer of anti‐ADAMTS13 IgG antibodies to distinguish iTTP patients with or without ADAMTS13 recovery**.
Sensitivity and specificity are shown at the optimal diagnostic cut‐off of anti‐ADAMTS13 IgG antibodies titer = 90.5 U/mL. Abbreviation: ADAMTS13: A Desintegrin And Metalloproteinase with ThromboSpondin‐1 motifs, 13rd member.

**Figure S3. Cumulative incidence curves of patients with ADAMTS13 recovery according to antibodies titer at baseline**.
Abbreviations: Abs: antibodies; ADAMTS13: A Desintegrin And Metalloproteinase with ThromboSpondin‐1 motifs, 13rd member; TPE: therapeutic plasma exchange.

**Figure S4. ADAMTS13 recovery at day 30 post‐TPE cessation according to the evolution of anti‐ADAMTS13 IgG antibodies titer in the validation cohort**.
Proportions of iTTP patients with ADAMTS13 recovery at day 30 post‐TPE cessation according to the evolution of anti‐ADAMTS13 IgG antibodies titer in the validation cohort (A). Cumulative incidence curve of patients with ADAMTS13 recovery according to the evolution of anti‐ADAMTS13 IgG antibodies titer in the validation cohort (B). T_0_ corresponds to day 14 post‐TPE cessation.
Abbreviations: ADAMTS13: A Desintegrin And Metalloproteinase with ThromboSpondin‐1 motifs, 13rd member; Dec + and Dec‐ denotes patients who decreased (+) or not (−) their anti‐ADAMTS13 IgG antibodies titer between diagnosis and day‐7 to day‐14 post‐TPE; TPE: therapeutic plasma exchange.

**Data S1.**Supporting Information.

## Data Availability

Data are available upon reasonable request (email paul.coppo@aphp.fr) and will be shared as in compliance with the General Data Protection Regulation and European Union privacy laws.
