# Supplementary figures and images for: Anti‐ADAMTS13 Antibodies Trajectory is Associated With ADAMTS13 Recovery in Immune‐Mediated TTP
Source: Am J Hematol. 2025 Jul 15;100(10):1736–46. doi: 10.1002/ajh.70005 (PMC12417756; doi:10.1002/ajh.70005)

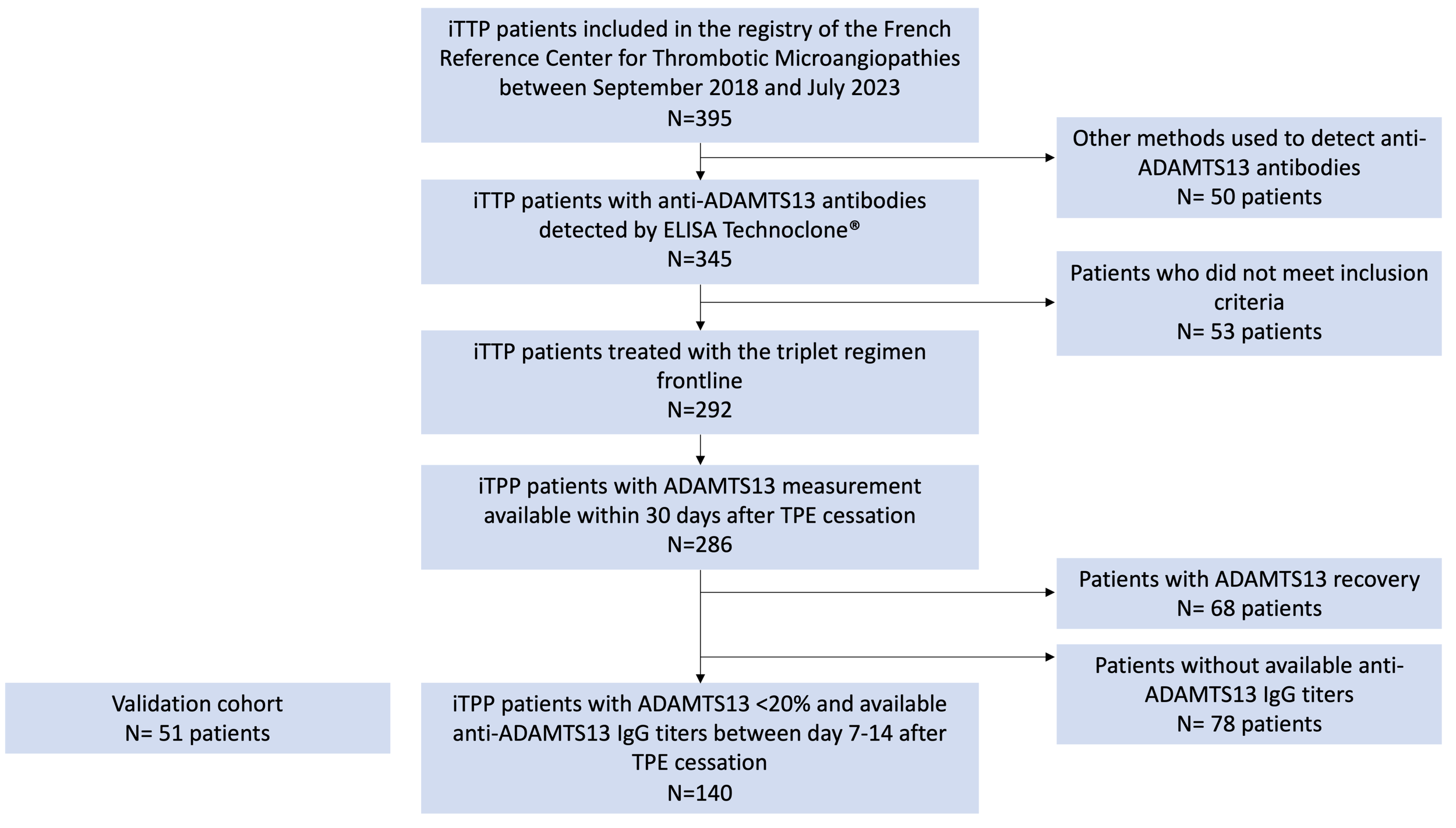

Supplement: Supplementary file 1 — Figure S1. Study flowchart. Abbreviations: ADAMTS13: A Desintegrin And Metalloproteinase with ThromboSpondin‐1 motifs, 13rd member; iTTP: immune‐mediated thrombotic thrombocytopenic purpura; TPE: therapeutic plasma exchange. *Three patients were not treated with TPE, 4 patients had an ADAMTS13 activity > 10%, and 46 patients had anti‐ADAMT13 IgG antibodies titer < 15 U/mL. [file AJH-100-1736-s004.tiff]

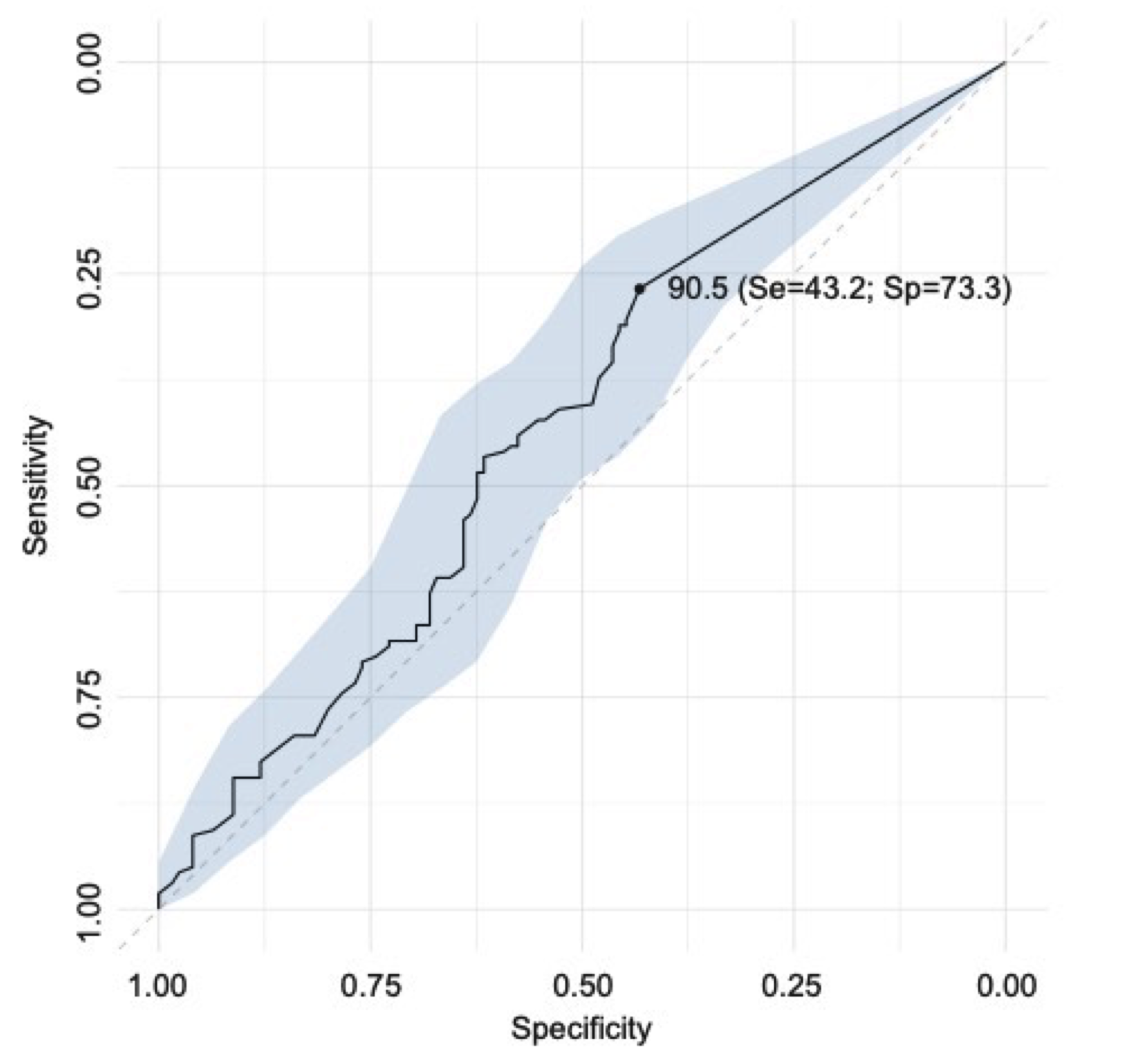

Supplement: Supplementary file 2 — Figure S2. ROC (Receiver Operating Characteristic) curve showing diagnosis accuracy of initial titer of anti‐ADAMTS13 IgG antibodies to distinguish iTTP patients with or without ADAMTS13 recovery. Sensitivity and specificity are shown at the optimal diagnostic cut‐off of anti‐ADAMTS13 IgG antibodies titer = 90.5 U/mL. Abbreviation: ADAMTS13: A Desintegrin And Metalloproteinase with ThromboSpondin‐1 motifs, 13rd member. [file AJH-100-1736-s002.tiff]

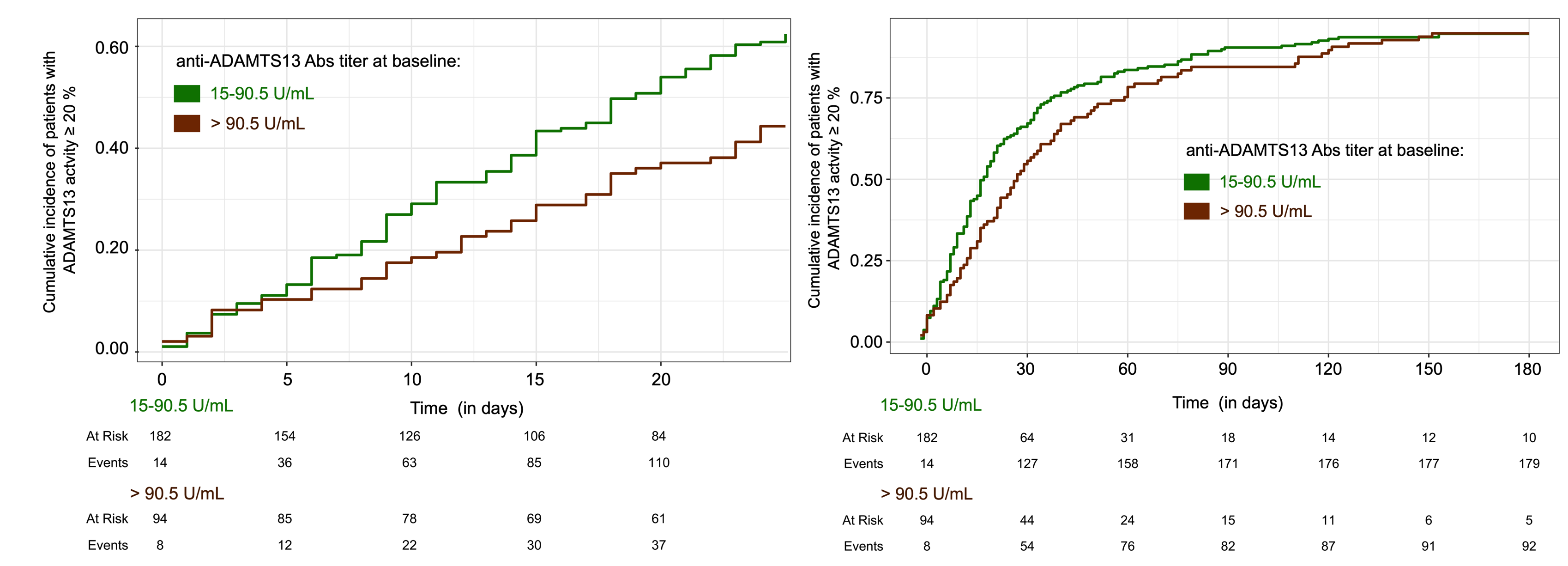

Supplement: Supplementary file 3 — Figure S3. Cumulative incidence curves of patients with ADAMTS13 recovery according to antibodies titer at baseline. Abbreviations: Abs: antibodies; ADAMTS13: A Desintegrin And Metalloproteinase with ThromboSpondin‐1 motifs, 13rd member; TPE: therapeutic plasma exchange. [file AJH-100-1736-s003.tiff]

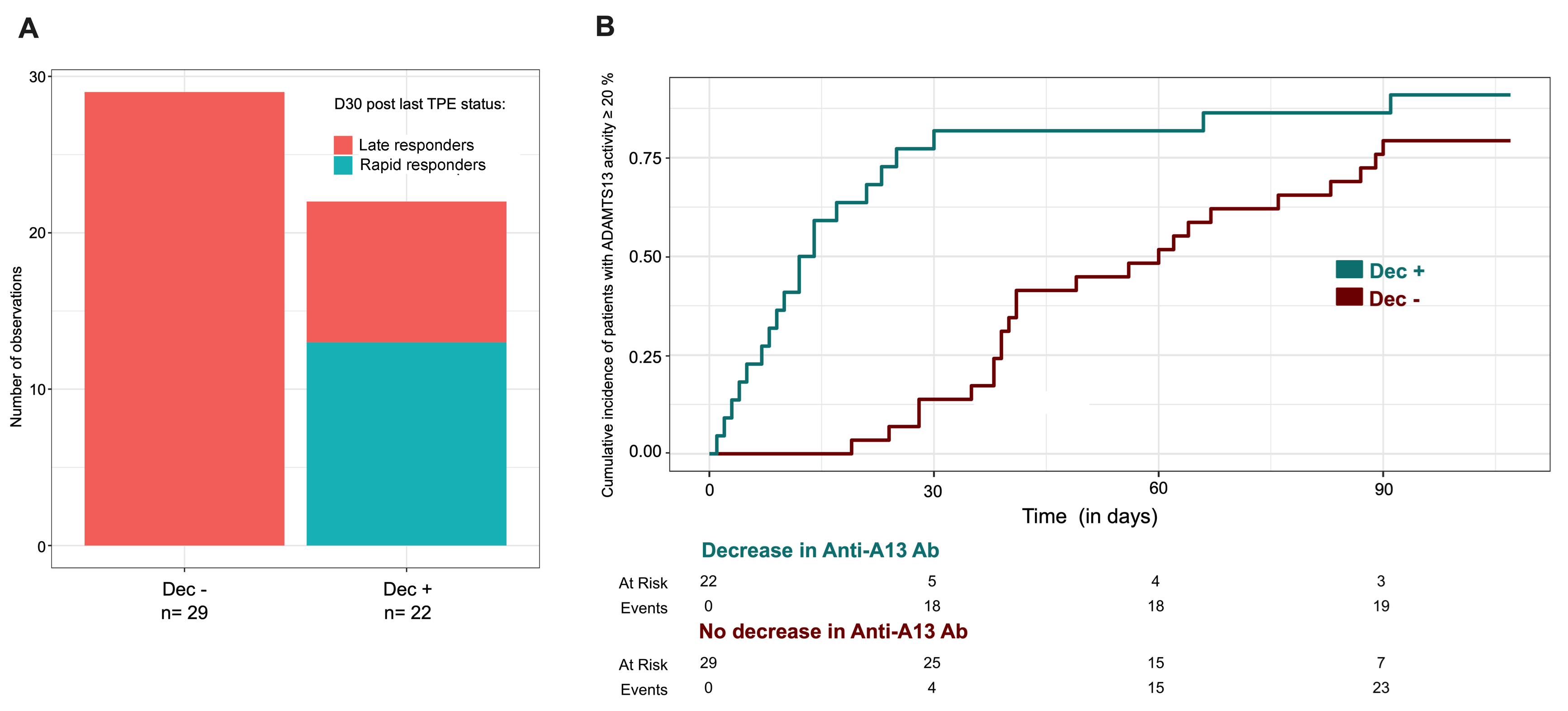

Supplement: Supplementary file 4 — Figure S4. ADAMTS13 recovery at day 30 post‐TPE cessation according to the evolution of anti‐ADAMTS13 IgG antibodies titer in the validation cohort. Proportions of iTTP patients with ADAMTS13 recovery at day 30 post‐TPE cessation according to the evolution of anti‐ADAMTS13 IgG antibodies titer in the validation cohort (A). Cumulative incidence curve of patients with ADAMTS13 recovery according to the evolution of anti‐ADAMTS13 IgG antibodies titer in the validation cohort (B). T0 corresponds to day 14 post‐TPE cessation. Abbreviations: ADAMTS13: A Desintegrin And Metalloproteinase with ThromboSpondin‐1 motifs, 13rd member; Dec + and Dec‐ denotes patients who decreased (+) or not (−) their anti‐ADAMTS13 IgG antibodies titer between diagnosis and day‐7 to day‐14 post‐TPE; TPE: therapeutic plasma exchange. [file AJH-100-1736-s001.tif]
