# Supplementary material for: Anti‐ADAMTS13 Antibodies Trajectory is Associated With ADAMTS13 Recovery in Immune‐Mediated TTP
Source: Am J Hematol. 2025 Jul 15;100(10):1736–46. doi: 10.1002/ajh.70005 (PMC12417756; doi:10.1002/ajh.70005)
Supplement: Supplementary file 5 — Data S1.Supporting Information. [file AJH-100-1736-s005.docx]

**Supplemental Data**

**Supplemental Table 1.** Clinical features at baseline and outcomes according to antibodies titer.

|  | Anti-ADAMTS13 antibodies titer ≤ 90.5 U/mL (N = 189) | Anti-ADAMTS13 antibodies titer > 90.5 U/mL (N = 97) | *p* |
| --- | --- | --- | --- |
| **Characteristics at baseline** |  |  |  |
| Age (y) | 42 [33–54] | 45 [31–60] | 0.5 |
| Female sex | 132 (70) | 61 (63) | 0.2 |
| Body mass index | 26.4 [22.7–32.2] | 26.9 [23.1–30.4] | 0.8 |
| Ethnicity |  |  | 0.2 |
| White | 139 (74) | 74 (78) |  |
| African-West Indies | 43 (23) | 15 (16) |  |
| Asian | 6 (3.2) | 6 (6.3) |  |
| History of iTTP | 25 (13) | 6 (6.3) | 0.074 |
| Cardiac involvement | 74 (39) | 28 (29) | 0.086 |
| Neurologic involvement | 124 (66) | 65 (67) | 0.9 |
| Platelet count (x10^3^/µL) | 12 [8–19] | 11 [8–18] | 0.5 |
| Hemoglobin (g/dL) | 8.3 [6.9–9.7] | 8.4 [6.8–9.8] | >0.9 |
| Serum creatinine level (µmol/L) | 89 [71–115] | 104 [72–136] | 0.03 |
| LDH level xN (U/L) | 3.8 [2.4–5.6] | 5 [3–6.4] | 0.042 |
| ADAMTS13 activity (%) | <10 | <10 | - |
| Anti-ADAMTS13 Abs titer (U/mL) | 53 [35–72] | 128 [100–221] | <0.001 |
| French Severity Score |  |  |  |
| Low | 124 (66) | 16 (16) | 0.2 |
| Intermediate | 45 (24) | 28 (29) |  |
| High | 20 (11) | 16 (16) |  |
|  |  |  |  |
| **Treatment** |  |  |  |
| TPE sessions to clinical response (days) | 4 [4–6] | 5 [4–7] | 0.03 |
| Corticosteroids | 189 (100) | 97 (100) | 0.6 |
| Rituximab | 189 (100) | 97 (100) | 0.6 |
| Number of rituximab infusion |  |  | 0.033 |
| <4 | 43 (23) | 15 (15) |  |
| 4 | 137 (72) | 70 (72) |  |
| >4 | 9 (4.8) | 12 (13) |  |
| Caplacizumab post-TPE (days) | 30 [23–37] | 30.5 [25.3–56.5] | 0.01 |
|  |  |  |  |
| **Outcome** |  |  |  |
| Clinical response | 187 (99) | 95 (99) | >0.9 |
| Time to clinical response from first TPE (days) | 4 [3–5] | 4 [3–7] | 0.006 |
| Refractoriness | 1 (0.5) | 1 (1) | >0.9 |
| 3-month death rate | 3 (2.2) | 2 (1.3) | >0.9 |
| Exacerbation under caplacizumab | 10 (5.3) | 5 (5.2) | >0.9 |
| Salvage therapy | 2 (1.1) | 1 (1) | >0.9 |
| Time from baseline to ADAMTS13 ≥20% (days) | 27 [17.3–45.8] | 37 [22–61.5] | 0.013 |
| ADAMTS13 activity ≥20% <30 days post- TPE (%) | 118 (62) | 43 (44) | 0.003 |
| Time to ADAMTS13 ≥20% post-TPE (days) | 23 [14–40] | 33 [19–60] | 0.004 |

Abbreviations: ADAMTS13: A Desintegrin And Metalloproteinase with ThromboSpondin-1 motifs, 13rd member; TPE: therapeutic plasma exchange; iTTP: immune-mediated thrombotic thrombocytopenic purpura; LDH: lactate dehydrogenase. Data are given as median (25^th^–75^th^ percentile) for quantitative variables and as n (%) for qualitative variables. Severe ADAMTS13 deficiency was defined as an activity < 10% (normal range for ADAMTS13 activity 50%-100%). The positivity threshold of anti-ADAMTS13 antibodies (Abs) was 15 U/mL, according to the manufacturer’s instructions (Technoclone^®^). Cardiac involvement was defined as the presence of clinical manifestations (e.g., chest pain) and/or electrocardiographic abnormalities. Neurologic involvement included the occurrence of confusion and/or stupor and/or seizures and/or coma and/or focal deficiency. Patients at high risk of early death of iTTP were defined by a French severity score ≥ 3 (cerebral involvement: yes = 1/no = 0, LDH: >10xULN = 1/≤10xULN = 0, age: >60 y = 2/<40 and ≤60 y = 1/≤40 y = 0). Cerebral involvement included confusion, stupor, coma or focal deficiency [1]. Clinical response corresponds to platelet count recovery (≥ 150x10^3^/µL).

**Supplemental Table 2.** Univariable and multivariable analysis of anti-ADAMTS13 IgG antibodies trajectory for ADAMTS13 recovery.

|  | Univariate | | Multivariable | |
| --- | --- | --- | --- | --- |
| Variables | OR [95% CI] | *p* | OR [95% CI] | *p* |
| Dec+ | 5.77 [2.79-12.39] | <0.001 | 7.24 [3.30-16.7] | <0.001 |
| Age | 0.99 [0.97-1.01] | 0.41 | 1.00 [0.96-1.03] | 0.86 |
| French Severity Score |  |  |  |  |
| Low | Reference | - | Reference | - |
| Intermediate | 0.81 [0.37-1.74] | 0.60 | 1.03 [0.36-2.98] | 0.95 |
| High | 0.77 [0.26-2.13] | 0.62 | 0.78 [0.15-3.89] | 0.76 |
| LDH level xN (U/L) | 0.99 [NA-1] | 0.66 | 1.00 [NA-1] | 0.70 |

Abbreviations: ADAMTS13: A Disintegrin And Metalloproteinase with ThromboSpondin-1 motifs, 13rd member; LDH: lactate dehydrogenase; OR: odds ratio; CI: confidence interval. Dec+ denote patients who decreased (+) their anti-ADAMTS13 antibodies titer between diagnosis and day-7 to day-14 post- therapeutic plasma exchange.

Patients at high risk of early death of iTTP were defined by a French severity score ≥ 3 (cerebral involvement: yes = 1/no = 0, LDH: >10xULN = 1/≤10xULN = 0, age: >60 y = 2/<40 and ≤60 y = 1/≤40 y = 0). Cerebral involvement included confusion, stupor, coma or focal deficiency [1]

**Supplemental Table 3.** Clinical features at baseline of the validation cohort according to anti-ADAMTS13 antibodies titer trajectory.

|  | Dec- group (N = 29) | Dec+ group (N = 22) | *p* |
| --- | --- | --- | --- |
| **Characteristics at baseline** |  |  |  |
| Age (y) | 43 [32- 54] | 41 [33.3–61.3] | >0.9 |
| Female Sex | 25 (86) | 12 (55) | 0.012 |
| Body mass index | 26.8 [24.2–31.1] | 28.1 [23.1–32.6] | >0.9 |
| Ethnicity |  |  |  |
| White | 24 (86) | 16 (73) | 0.5 |
| African-West Indies | 3 (11) | 4 (18) |  |
| Asian | 1 (3.6) | 2 (9.1) |  |
| History of iTTP | 6 (21) | 0 | 0.031 |
| Cardiac involvement | 4 (25) | 5 (29) | >0.9 |
| Neurological involvement | 6 (24) | 1 (4.5) | 0.10 |
| Platelet count (x10^3^/µL) | 13 [8–21] | 10 [8.3–18.8] | 0.9 |
| Hemoglobin (g/dL) | 8.8 [7.5–9.6] | 7.9 [6.6–9.3] | 0.2 |
| Serum creatinine level (µmol/L) | 95 [77–124] | 92 [79–114] | >0.9 |
| LDH level xN (U/L) | 3.4 [2–5] | 4 [2.5–6.3] | 0.3 |
| ADAMTS13 activity (%) | <10 | <10 | - |
| Anti-ADAMTS13 Abs titer (U/mL) | 79 [45.3–150] | 81 [60–150] | 0.8 |
| French Severity Score |  |  | 0.3 |
| Low | 24 (86) | 15 (68) |  |
| Intermediate | 3 (11) | 5 (23) |  |
| High | 1 (3.6) | 2 (9.1) |  |
|  |  |  |  |
| **Treatment** |  |  |  |
| TPE sessions to clinical response | 3 [3–4.5] | 4 [3–6] | 0.13 |
| Corticosteroids | 29 (100) | 22 (100) | - |
| Rituximab | 29 (100) | 22 (100) | - |
| Number of rituximab infusion |  |  | 0.3 |
| <4 infusions | 4 (14) | 1 (4.5) |  |
| 4 infusions | 22 (79) | 21 (95) |  |
| >4 infusions | 2 (7.1) | 0 |  |
| Caplacizumab post-TPE (days) | 58 [42.8–77.3] | 36 [27.3–53.3] | 0.035 |
| Abbreviations: ADAMTS13: A Disintegrin And Metalloproteinase with ThromboSpondin-1 motifs, 13rd member; TPE: therapeutic plasma exchange; iTTP: immune-mediated thrombotic thrombocytopenic purpura; LDH: lactate dehydrogenase; Dec+ and Dec- denotes patients who decreased (+) or not (-) their anti-ADAMTS13 antibodies titer between diagnosis and day-7 to day-14 post-TPE. Data are given as median (25^th^–75^th^ percentile) for quantitative variables and as n (%) for qualitative variables. Severe ADAMTS13 deficiency was defined as an activity < 10% (normal range for ADAMTS13 activity 50%-100%). The positivity threshold of anti-ADAMTS13 antibodies (Abs) was 15 U/mL, according to the manufacturer’s instructions (Technoclone^®^). Cardiac involvement was defined as the presence of clinical manifestations (e.g., chest pain) and/or electrocardiographic abnormalities. Neurologic involvement included the occurrence of confusion and/or stupor and/or seizures and/or coma and/or focal deficiency. Patients at high risk of early death of iTTP were defined by a French severity score ≥ 3 (cerebral involvement: yes = 1/no = 0, LDH: >10xULN = 1/≤10xULN = 0, age: >60 y = 2/<40 and ≤60 y = 1/≤40 y = 0). Cerebral involvement included confusion, stupor, coma or focal deficiency [1]. | | | |

**Supplemental Figure Legend**

**Supplemental Figure 1. Study flowchart.**

Abbreviations: ADAMTS13: A Desintegrin And Metalloproteinase with ThromboSpondin-1 motifs, 13rd member; TPE: therapeutic plasma exchange; iTTP: immune-mediated thrombotic thrombocytopenic purpura. *Three patients were not treated with TPE, 4 patients had an ADAMTS13 activity >10%, and 46 patients had anti-ADAMT13 antibodies titer <15 U/mL.

**Supplemental Figure 2. ROC (Receiver Operating Characteristic) curve showing diagnosis accuracy of initial titer of anti-ADAMTS13 IgG antibodies to distinguish iTTP patients with or without ADAMTS13 recovery.**

Sensitivity and specificity are shown at the optimal diagnostic cut-off of anti-ADAMTS13 antibodies titer = 90.5 U/mL. Abbreviation: ADAMTS13: A Desintegrin And Metalloproteinase with ThromboSpondin-1 motifs, 13rd member.

**Supplemental Figure 3. Cumulative incidence curves of patients with ADAMTS13 recovery according to antibodies titer at baseline.**

Abbreviations: ADAMTS13: A Desintegrin And Metalloproteinase with ThromboSpondin-1 motifs, 13rd member; TPE: therapeutic plasma exchange; Abs: antibodies.

**Supplementary Figure 4. ADAMTS13 recovery at day 30 post-TPE cessation according to the evolution of anti-ADAMTS13 antibodies titer in the validation cohort.**

Proportions of iTTP patients with ADAMTS13 recovery at day 30 post-TPE cessation according to the evolution of anti-ADAMTS13 antibodies titer in the validation cohort (A). Cumulative incidence curve of patients with ADAMTS13 recovery according to the evolution of anti-ADAMTS13 antibodies titer in the validation cohort (B). T_0_ corresponds to day 14 post-TPE cessation.

Abbreviations: ADAMTS13: A Desintegrin And Metalloproteinase with ThromboSpondin-1 motifs, 13rd member; TPE: therapeutic plasma exchange; Dec+ and Dec- denotes patients who decreased (+) or not (-) their anti-ADAMTS13 antibodies titer between diagnosis and day-7 to day-14 post-TPE.

**Appendix**

**The members of the Reference Center for Thrombotic Microangiopathies (CNR-MAT) are:** Augusto Jean-François (Service de Néphrologie, dialyse et transplantation ; CHU Larrey, Angers); Azoulay Elie (Service de Réanimation Médicale, Hôpital Saint-Louis, Paris); Barbay Virginie (Laboratoire d’Hématologie, CHU Charles Nicolle, Rouen); Benhamou Ygal (Service de Médecine Interne, CHU Charles Nicolle, Rouen); Charasse Christophe (Service de Néphrologie, Centre Hospitalier de Saint-Brieuc); Charvet-Rumpler Anne (Service d’Hématologie, CHU de Dijon) ; Chauveau Dominique, Ribes Davis (Service de Néphrologie et Immunologie Clinique, CHU Rangueil, Toulouse); Choukroun Gabriel (Service de Néphrologie, Hôpital Sud, Amiens); Coindre Jean-Philippe (Service de Néphrologie, CH Le Mans); Coppo Paul (Service d’Hématologie, Hôpital Saint-Antoine, Paris); Delmas Yahsou (Service de Néphrologie, CHU de Bordeaux, Bordeaux); Kwon Theresa (Service de Néphrologie Pédiatrique, Hôpital Robert Debré, Paris); Salanoubat Célia (Service d’Hématologie, Hôpital Sud-Francilien, Corbeil-Essonnes); Dossier Antoine (Service de Médecine Interne, Hôpital Bichat, Paris); Fain Olivier (Service de Médecine Interne, Hôpital Saint-Antoine, Paris); Ville Simon (Service de Néphrologie, CHU Hôtel-Dieu, Nantes) ; Frémeaux-Bacchi Véronique (Laboratoire d’Immunologie, Hôpital Européen Georges Pompidou, Paris); Galicier Lionel (Service d’Immunopathologie, Hôpital Saint-Louis, Paris); Grangé Steven (Service de Réanimation Médicale, CHU Charles Nicolle, Rouen) ; Guidet Bertrand (Service de Réanimation Médicale, Hôpital Saint-Antoine, Paris); Halimi Jean-Michel (Service de Néphrologie Pédiatrique, Hôpital Bretonneau, Tours); Hamidou Mohamed, Neel Antoine (Service de Médecine Interne, Hôtel-Dieu, Nantes); Fornecker Luc-Matthieu (service d’Oncologie et d’Hématologie, Hôpital de Hautepierre, Strasbourg); Hié Miguel (Service de Médecine Interne, Groupe Hospitalier Pitié-Salpétrière, Paris) ; Jacobs Frédéric (Service de Réanimation Médicale, Hôpital Antoine Béclère, Clamart); Joly Bérangère (Service d’Hématologie Biologique, Hôpital Lariboisière, Paris) ; Kanouni Tarik (Unité d’Hémaphrèse, Service d’Hématologie, CHU de Montpellier) ; Kaplanski Gilles (Service de Médecine Interne, Hôpital la Conception, Marseille) ; Rieu Claire (Hôpital d’Estaing, Service de Médecine Interne, Clermont-Ferrand); Le Guern Véronique (Unité d’Hémaphérèse, Service de Médecine Interne, Hôpital Cochin, Paris) ; Moulin Bruno (Service de Néphrologie, Hôpital Civil, Strasbourg); Rebibou Jean-Michel (Service de Néphrologie, CHU de Dijon); Ojeda Uribe Mario (Service d’Hématologie, Hôpital Emile Muller, Mulhouse); Parquet Nathalie (Unité de Clinique Transfusionnelle, Hôpital Cochin, Paris); Pène Frédéric (Service de Réanimation Médicale, Hôpital Cochin, Paris) ; Perez Pierre (Service de Réanimation polyvalente, CHU de Nancy) ; Poullin Pascale (Service d’hémaphérèse et d’autotransfusion, Hôpital la Timone, Marseille); Marie Manon (Service de Médecine Interne, CHU Édouard Herriot, Lyon); Presne Claire (Service de Néphrologie, Hôpital Nord, Amiens); Provôt François (Service de Néphrologie, Hôpital Albert Calmette, Lille); Mesnard Laurent (Urgences Néphrologiques et Transplantation Rénale, Hôpital Tenon, Paris); Saheb Samir (Unité d’Hémaphérèse, Hôpital la Pitié-Salpétrière, Paris) ; Seguin Amélie (Service de Réanimation Médicale, CHU Hôtel-Dieu, Nantes) ; Servais Aude (Service de Néphrologie, CHU Necker-Enfants Malades) ; Stépanian Alain (Laboratoire d’Hématologie, Hôpital Lariboisière, Paris); Veyradier Agnès (Service d’Hématologie Biologique, Hôpital Lariboisière, Paris); Vigneau Cécile (Service de Néphrologie, Hôpital Pontchaillou, Rennes); Wynckel Alain (Service de Néphrologie, Hôpital Maison Blanche, Reims); Zunic Patricia (Service d’Hématologie, Groupe Hospitalier Sud-Réunion, la Réunion).

**References**

1. Benhamou Y, Assie C, Boelle PY, et al. Development and validation of a predictive model for death in acquired severe ADAMTS13 deficiency-associated idiopathic thrombotic thrombocytopenic purpura: the French TMA Reference Center experience. *Haematologica*. Aug 2012;97(8):1181-6. doi:10.3324/haematol.2011.049676
